# Computational and synthetic studies towards improving rescinnamine as an inducer of MSH2-dependent apoptosis in cancer treatment

**Published:** 2013

**Authors:** ElShimaa M. N. AbdelHafez, Andrew Diamanduros, Lacramioara Negureanu, Yan Luy, J. Hayley Bean, Katherine Zielke, Brittany Crowe, Aksana Vasilyeva, Jill E. Clodfelter, Omar M. Aly, Gamal El-Din A. A. Abuo-Rahma, Karin D. Scarpinato, Freddie R. Salsbury, S. Bruce King

**Affiliations:** 1 Department of Chemistry, Wake Forest University, Winston-Salem, NC; 2 Department of Biology, Georgia Southern University, Statesboro, GA; 3 Department of Cancer Biology, Wake Forest University School of Medicine, Winston-Salem, NC; now: St. Jude Hospital, Memphis, TN; 4 Department of Biochemistry, Wake Forest University School of Medicine, Winston-Salem, NC; 5 Department of Physics, Wake Forest University, Winston-Salem, NC; 6 Department of Medicinal Chemistry, Faculty of Pharmacy, Minia University, Minia , Egypt

## Abstract

We, and others, have previously shown that mismatch repair proteins, in addition to their repair function, contribute to cell death initiation. In response to some drugs, this cell death activity is independent of the repair function of the proteins. Rescinnamine, a derivative of the indole alkaloid reserpine, a drug used to treat hypertension several decades ago, was shown to target the cell death-initiating activity of mismatch repair proteins. When used in animals, the hypotensive action of this drug prevents applying appropriate concentrations for statistically significant tumor reduction. Using a combination of computational modeling, chemical synthesis and cell assays, we determine how rescinnamine can be structurally modified and what effect these modifications have on cell survival. These results inform further computational modeling to suggest new synthetic lead molecules to move toward further biological testing.

## INTRODUCTION

Mismatch repair proteins function in a number of cellular processes including cytotoxic response and apoptosis. The precise mechanism of their involvement in apoptosis remains to be determined and appears to be dependent on the type of damage to the cell (Topping et al., 2009). We previously used computational modeling to identify a distinct, death-inducing conformation of the mismatch repair proteins MSH2/MSH6 (Salsbury et al., 2006) that can be targeted for the treatment of cancer cells (Vasilyeva et al., 2009, 2010). The MSH2/MSH6-dependent cell death pathway activates pro-apoptotic proteins, such as caspase-3 and caspase-9, but is independent of many proteins commonly mutated in cancer, e.g. p53 (Topping et al., 2009).

Our initial computational approach that examined two thousand possible compounds targeting the MSH2/MSH6 death conformation identified two Rauwolfia alkaloids, reserpine and rescinnamine that are expected to mimic the response induced by cisplatin (Vasilyeva et al., 2009). Rescinnamine showed the best cytotoxic activity by activating pro-apoptotic proteins (Vasilyeva et al., 2010). It is a member of an understudied group of compounds previously used as anti-hypertensive drugs in the 1950s and 60s that target the angiotensin-converting enzyme (ACE). Rescinnamine has lower neurotoxicity, but also lower antihypertensive efficacy than reserpine (Fife et al., 1960). The drug has no known genotoxic or chromosome destabilizing effects (Brambilla & Martinelli, 2006). Neither one of these compounds had been systematically studied as anticancer agents.

Here, we demonstrate that the anti-hypertensive effects limit the usefulness of rescinnamine as a tumorinhibiting drug, and employ computational simulation and organic synthesis to modify the compound to develop more efficient anti-cancer drugs. Application of the palladium-mediated Heck coupling reaction to this structurally complicated alkaloid natural product allows for the efficient preparation of rescinnamne derivatives to test computational predictions and build an initial structure-activity relationship (SAR). Computationally, we extend our successful protocol of using structures obtained from molecular dynamics simulations along with docking into structures representative of the death-inducing conformations (Vasilyeva et al., 2009). However, we have improved upon our previous methods in three ways. First, we used structures obtained via cluster analysis of a longer-time scale simulation of hMSH2/6 (Negureanu and Salsbury, 2012). Second, we upgraded to Autodock4 (Morris et al, 2009), with its improved force field, from Autodock3 (Morris et al, 1998). Third, we focused on modification of reserpine and rescinnamine, rather than docking new compounds, which required development of a library of modified compounds that were then docked and ranked.

## MATERIALS AND METHODS

### Cell Biology

HEC59 cells (msh2 deficient) and the paired cell line HEC59 chr. 2 that restores the MSH2-deficiency via chromosome transfer have been extensively characterized (Umar et al., 1997). Cells were grown in standard growth media (DMEM-F12 + 10% FBS). Cells were plated in microtiter plates at an appropriate concentration in 100 µl media and incubated overnight. Media was replaced with media containing drug and allowed to incubate for 24 hours at indicated concentrations. Untreated cells received fresh media with vehicle only. One solution reagent (CellTiter 96)(r) Aqueous One Solution) was added to existing media (20 µl/well) and allowed to incubate 3–4 hrs. A plate reader was used to record the absorbance at 490 nm. Assays were performed at least in triplicates. Cell viability at each concentration was analyzed for IC_50_ values using GraphPad Prism 4™. Graphs represent mean values and standard deviations.

### Xenograft

SW416 or HEC59 cells in PBS mixed with Matrigel (1:1; BD Biosciences) were subcutaneously injected into the flank of nude donor mice. Tumors were grown for up to 3 weeks. Mice were euthanized, tumors excised, minced into 3 mm pieces, and surgically implanted into the right flank of acceptor mice (10 per group). Isoflurane anesthesia was provided during tumor inoculation. Injection of compounds was started 3 days following tumor implantation to allow recovery from surgery. Compounds were given intraperitoneally, in a volume of 0.5 ml/mouse with a ¼ inch, 23-gauge needle in a 6 ml plastic syringe. Mice were monitored based on survival and body weight. Tumor size, measured by caliper, and body weight were monitored daily for 57 days, and the prolongation of median survival time after ip treatment determined. Any animals showing signs of distress, unnatural movements, severe loss of appetite, severe signs of hypotension, tumor size of 1000g, or weight loss exceeding 10% before the end of the study were euthanized. Tumors were measured twice a week for each group. Tumor volume is calculated as length (mm) x width (mm)^2^. Initial measurements were performed when the tumor reached 150–200 mg. Tumor weight (in mg) is calculated as tumor weight (mg) - (length(mm) of tumor x width (mm) of tumor^2^)/2.

### Chemistry-General

Reagents were obtained from commercial sources and used without additional purification. Extraction and flash chromatography solvents were technical grade. Flash chromatography was carried out using a Biotage SP1-B2A0/ HPFC System. Analytical thin layer chromatography (TLC) was performed on silica gel plates with C-4 Spectroline 254 indicator. Visualization was accomplished with UV light and 20% phosphomolybdic acid solution in EtOH. LC-MS, ESI-MS and HPLC solvents were HPLC grade. Melting points were determined on a Mel-Temp apparatus. ^1^H NMR and ^13^CNMR spectra were taken in commercial deuterated solvents and recorded on a Bruker Advance 300 MHz and Bruker DRX-500 spectrometer using a 5mm TBI probe equipped with z axis gradients. Probe temperature was regulated at 25° C. All data was collected and processed with Topspin 1.3 using standard Bruker processing parameters. Chemical shifts (δ) are given in ppm; multiplicities are indicated by s (singlet), d (doublet), t (triplet), q (quartet), m (multiplet), dd (doublet of doublet) and br (broad).

### Acryloyl reserpate (1)

Dry distilled pyridine (791mg, 0.808mL, 10mmole) was added to a mixture of methyl reserpate (300mg, 0.74mmole) and acryloyl chloride (135.77mg, 0.123mL, 1.5mmole) was added and stirred under nitrogen at room temperature for 74 hr. Excess pyridine was evaporated and the residue was taken up in chloroform (50 mL) and the organic layer washed with water (3× 20 mL) and brine, the organic layer was dried with anhydrous Na_2_SO_4_, filtered and evaporated under reduced pressure to give a residue that was purified by flash chromatography (silica gel, chloroform/methanol, 3%, R_f_ 0.3) giving a solid rinsed with methanol to afford 1 (120 mg, 35%) as white crystals : mp >260 °C; ^1^H-NMR (300 MHz, CDCl_3_, δ): 7.80 (s, 1H), 7.31(d, 1H, J= 8.52 Hz), 6.81 (s, 1H, J= 1.95 Hz), 6.76 (d, 1H, J= 8.53 Hz), 6.45(d, 1H, J= 17.32 Hz), 6.15 (dd, 1H, J= 17.31& 17.30 Hz), 5.87 (d, 1H, J= 10.4 Hz), 4.90-4.82 (m, 1H), 4.42 (s, 1H), 3.82 (s, 3H), 3.80 (s, 3H), 3.48 (s, 3H), 3.19-3.14 (m, 2H), 3.02-2.98 (m, 1H), 2.69- 2.60 (m, 2H), 2.49-2.39 (m,2 H), 2.26- 2.17 (m, 2H), 2.04-1.78 (m, 5H); ^13^C-NMR (75 MHz, CDCl_3_, δ): 172.77, 165.42, 156.10, 136.31, 130.79, 130.45, 128.63, 122.13, 118.45. 108.92, 107.92, 95.18, 77.69, 77.54, 60.68, 55.76, 53.68, 51.75, 51.72, 51.17, 48.96, 33.93, 32.25, 29.47, 24.17, 16.75; ESI-MS: m/z= 469.2 (M^+^ + H); Anal. Calcd. for C_26_H_32_N_2_O_6_.0.3H_2_O, C, 65.81; H, 6.94; N, 5.90. Found: C, 65.60; H, 6.83; N, 6.01.

### General procedure for the synthesis of rescinnamine derivatives (2–7)

A mixture of the aryl iodide (0.507 mmole), acryloyl reserpate (300 mg, 0.641 mmole), Et_3_N (0.06 g, 0.09 mL, 0.645 mmol) and Pd(OAc)_2_ (1.23 mg, 0.005mmole) in the presence of tri-o-tolylphosphine (4.5 mg, 0.021mmole) in acetonitrile (20 mL) was heated with stirring in a capped sealed glass tube under argon at 90°C for 48hrs. After cooling, the solvent was removed under reduced pressure; the residue was dissolved in chloroform (30 mL) and washed with water (3×15 mL) and brine. The organic layer was dried with anhydrous sodium sulfate, filtered and evaporated under reduced pressure to give a residue that was purified by flash chromatography to afford a solid that was recrystallized as described below:

#### For 2: (R_1_ = COOCH_3_, R_2_ = OCH_3_)

White crystals recrystallized from methanol, yield = 180 mg (44%), (chloroform/methanol 3%, R_f_ = 0.7), mp = 215–217 °C; ^1^H NMR (500 MHz, CDCl_3_, δ): 8.03 (s, 1H), 7.69 (s, 1H), 7.67-7.66 (m, 2H), 7.35 (d, 1H, J= 8.52), 7.02 (d, 1H, J= 8.77 Hz), 6.86 (s, 1H), 6.79 (d, 1H, J= 8.5 Hz), 6.40 (d, 1H, J= 15.98), 4.97-4.92 (m, 1H), 4.47 (s, 1H), 3.97 (s, 3H), 3.95 (s, 3H), 3.86(s, 3H), 3.84 (s, 3H), 3.54 (s, 3H), 3.19-3.18 (m, 2H), 3.06-3.03 (m, 1H), 2.69-2.66 (m, 1H), 2.52-2.45 (m, 3H), 2.30- 2.25 (m, 2H), 1.98-1.80 (m, 5H); ^13^C NMR (125 MHz, CDCl_3_, δ): 172.94, 166.41, 166.11, 160.58, 156.03, 143.66, 136.30, 133.49, 131.63, 130.43, 126.48, 122.05, 120.28, 118.55, 116.84, 112.34, 108.94, 107.85, 95.04, 65.93, 60.97, 56.26, 55.76, 53.69, 52.40, 51.76, 51.17, 48.92, 33.92, 32.25, 31.08, 29.62, 24.21, 16.75, 15.33; ESI-MS: m/z= 633.4 (M^+^ + H); Anal. Calcd for C_35_H_40_N_2_O_9_: C, 66.44; H, 6.37; N, 4.43; Found: C, 66.16; H, 6.46; N, 4.41.

#### For 3 (R_1_ = OCH_3_, R_2_ = COOH)

Yellow crystals recrystallized from diethyl ether, yield = 150 mg (38%), (chloroform/methanol 8%, R_f_ = 0.1), mp = 228–230 °C; ^1^H NMR (300 MHz, DMSO, δ): 10.62 (s, 1H), 7.70 (d, 1H, J= 15.97 Hz), 7.58 (d, 1H, J=7.83 Hz), 7.47 (s, 1H), 7.32 (d, 1H, J= 7.83 Hz), 7.24 (d, 1H, J= 8.55Hz), 6.82 (s, 1H), 6.79 (d, 1H, J= 15.91 Hz), 6.63 (d, 1H, J= 8.57 Hz), 4.90-4.82 (m, 1H), 4.53 (s, 1H), 3.88 (s, 3H), 3.79 (s, 3H), 3.75 (s, 3H), 3.43 (s, 3H), 3.17-3.11 (m, 3H), 2.97-2.84 (m, 3H), 2.72-2.66 (m, 1H), 2.24 (d, 1H), 1.87-1.73 (m, 6H); ^13^C NMR (75 MHz, DMSO, δ): 171.81, 170.22, 165.82, 157.10, 155.80, 144.75, 137.20, 136.09, 129.43, 128.36, 121.26, 120.57, 118.97, 118.67, 111.83, 108.99, 105.82, 94.88, 79.40, 77.59, 76.63, 60.55, 55.99, 55.40, 53.47, 52.29, 50.73, 50.06, 48.87, 32.21, 31.29, 28.94, 23.05, 16.00; ESI-MS: m/z= 619.3 (M^+^ + H); Anal. Calcd for C_3_4H_38_N_2_O_9_.2.5H_2_O: C, 61.53; H, 6.53; N: 4.22; Found: C, 61.41; H, 7.11; N, 4.00.

#### For 4 (R_1_ = COOH, R_2_ = OCH_3_)

Pale yellow solid recrystallized from diethy ether, yield = 108 mg (27%), (chloroform/methanol 8%, R_f_ = 0.1), mp = 249–250 °C; ^1^H NMR (500 MHz, DMSO, δ): 10.61 (s, 1H), 8.32 (s, 1H), 7.93 (s, 1H), 7.87 (d, 1H, J= 8.57 Hz), 7.70 (d, 1H, J= 15.96 Hz), 7.24 (d, 1H, J= 8.44 Hz), 7.16 (d, 1H, J= 8.66 Hz), 6.82 (s, 1H), 6.63 (d, 1H, J= 8.34 Hz), 6.55 (d, 1H, J= 15.99 Hz), 4.81-4.79 (m, 1H), 4.49 (s, 1H), 3.87 (s, 3H), 3.80 (s, 3H), 3.75 (s, 3H), 3.42 (s, 3H), 3.14-3.13 (m, 3H), 2.94-2.85 (m, 3H), 2.68-2.66 (m, 1H), 2.21-2.16 (m, 1H), 1.99-1.74 (m, 6H); ^13^C NMR (125 MHz, DMSO, δ): 172.07, 168.17, 166.19, 159.75, 155.79, 144.34, 137.13, 132.76, 130.86, 126.43, 123.99, 121.87, 118.53, 116.70, 113.15, 108.81, 106.24, 95.28, 79.59, 77.91, 77.02, 60.64, 56.41, 55.67, 53.97, 52.31, 51.31, 50.96, 48.69, 33.17, 32.12, 29.67, 23.67, 16.57; ESI-MS: m/z= 619.3 (M^+^ + H); Anal. Calcd for C_34_H_38_N_2_O_9_.3H_2_O: C, 60.70; H, 6.59; N, 4.16; Found: C, 60.76; H, 6.39; N, 4.07.

#### For 5 (R_1_ = OCH_3_, R_2_ = OH)

White flakes from chloroform, yield = 108 mg (27%), (chloroform/methanol 4%, R_f_ = 0.3), mp = 259–260 °C; ^1^H NMR (300 MHz, DMSO, δ): 10.49 (s, 1H), 9.61 (s, br, 1H), 7.62 (d, 1H, J= 15.87 Hz), 7.35 (s, 1H), 7.21 (d, 1H, J= 8.48 Hz), 7.14 (d, 1H, J= 8.35Hz), 6.82-6.79 (m, 2H), 6.61 (d, 1H, J= 8.49 Hz), 6.50 (d, 1H, J= 15.89 Hz), 4.87-4.78 (m, 1H), 4.33 (s, 1H), 3.83 (s, 3H), 3.78 (s, 3H), 3.75 (s, 3H), 3.41 (s, 3H), 3.04-3.01 (m, 2H), 2.87-2.81 (m, 2H), 2.66-2.63 (m, 1H), 2.36- 2.31 (m, 2H), 2.19-2.12 (m, 1H), 2.04- 2.00 (m, 1H), 1.93-1.69 (m, 5H); ^13^C NMR (75 MHz, DMSO, δ): 172.88, 166.49, 156.23, 148.72, 145.98, 144.79, 136.34, 130.55, 128.02, 122.22, 121.86, 118.55, 116.23, 113.18, 110.59, 109.05, 108.13, 95.20, 77.84, 65.84, 60.77, 55.99, 55.82, 53.71, 51.78, 51.19, 49.06, 34.04, 32.34, 29.69, 24.28, 16.81, 15.26; ESI-MS: m/z= 591.2 (M^+^ + H).

#### For 6 (R_1_ = OH, R_2_ = OCH_3_)

Yellow solid recrystallized from diethyl ether, yield = 174 mg (43%), (chloroform/methanol 3%, R_f_ = 0.3), mp = 180–182 °C; ^1^H NMR (300 MHz, CDCl_3_, δ): 7.77 (s, 1H), 7.63 (d, 1H, J= 15.92 Hz), 7.36 (d, 1H, J= 8.54 Hz), 7.32 (d, 1H, J= 8.54 Hz), 7.27 (s, 1H), 6.97 (d, 1H, J= 8.56 Hz), 6.82 (s, 1H), 6.76 (d, 1H, J= 8.53 Hz), 6.33 (d, 1H, J= 15.92 Hz), 4.95-4.87 (m, 1H), 4.42 (s, 1H), 3.85 (s, 3H), 3.82 (s, 3H), 3.81 (s, 3H), 3.50 (s, 3H), 3.49-3.44 (m, 2H), 3.18-3.14 (m, 2H), 3.02-2.97 (m, 1H), 2.67-2.62 (dd, 1H), 2.49-2.39 (m, 3H), 2.33 (s, 3H), 2.04-1.78 (m, 5H).; ^13^C NMR (125 MHz, CDCl_3_, δ): 172.86, 168.88, 166.34, 156.18, 152.97, 143.86, 140.06, 136.38, 130.57, 127.82, 127.50, 122.21, 121.94, 118.51, 116.76, 112.35, 108.98, 107.99, 95.25, 77.87, 65.84, 60.79, 56.02, 55.82, 53.74, 51.79, 51.23, 49.04, 34.03, 32.32, 29.69, 24.25, 20.62, 16.81, 15.27; ESI-MS: m/z= 6.33.3 (M^+^ + H); Anal. Calcd for C_35_H_40_N_2_O_9_.0.25H_2_O: C, 65.97; H, 6.41; N, 4.40; Found: C, 65.74; H, 6.55; N, 4.49.

#### For 7 (R_1_ = OCOCH_3_, R_2_ = OCH_3_)

Yellow solid recrystallized from diethyl ether, yield = 174 mg (43%), (chloroform/methanol 3%, R_f_ = 0.3), mp = 180–182 °C; ^1^H NMR (300 MHz, CDCl_3_, δ): 7.77 (s, 1H), 7.63 (d, 1H, J= 15.92 Hz), 7.36 (d, 1H, J= 8.54 Hz), 7.32 (d, 1H, J= 8.54 Hz), 7.27 (s, 1H), 6.97 (d, 1H, J= 8.56 Hz), 6.82 (s, 1H), 6.76 (d, 1H, J= 8.53 Hz), 6.33 (d, 1H, J= 15.92 Hz), 4.95-4.87 (m, 1H), 4.42 (s, 1H), 3.85 (s, 3H), 3.82 (s, 3H), 3.81 (s, 3H), 3.50 (s, 3H), 3.49-3.44 (m, 2H), 3.18-3.14 (m, 2H), 3.02-2.97 (m, 1H), 2.67-2.62 (dd, 1H), 2.49-2.39 (m, 3H), 2.33 (s, 3H), 2.04-1.78 (m, 5H).; ^13^C NMR (125 MHz, CDCl_3_, δ): 172.86, 168.88, 166.34, 156.18, 152.97, 143.86, 140.06, 136.38, 130.57, 127.82, 127.50, 122.21, 121.94, 118.51, 116.76, 112.35, 108.98, 107.99, 95.25, 77.87, 65.84, 60.79, 56.02, 55.82, 53.74, 51.79, 51.23, 49.04, 34.03, 32.32, 29.69, 24.25, 20.62, 16.81, 15.27; ESI-MS: m/z= 6.33.3 (M^+^ + H); Anal. Calcd for C_35_H_40_N_2_O_9_.0.25H_2_O: C, 65.97; H, 6.41; N, 4.40; Found: C, 65.74; H, 6.55; N, 4.49.

#### Propionoyl reserpate (8)

Using the same procedure for 1 and substituting propionyl chloride for acryloyl chloride yields a residue that was purified by flash chromatography (silica gel, chloroform/methanol, 3%, R_f_ 0.2) to give a solid that was rinsed with methanol/diethylether (1:1) to afford 8 (317 mg, 56%) as a fine light yellow powder : mp = 258 °C; ^1^H-NMR (300 MHz, CDCl_3_, δ): 7.60 (br, s, 1H), 7.32 (d, 1H, J= 8.54 Hz), 6.82 (s, 1H), 6.76 (d, 1H, J= 8.59 Hz), 4.81-4.72 (m, 1H), 4.41 (s, 1H), 3.83 (s, 3H), 3.80 (s, 3H), 3.74-3.71 (m, 1H), 3.49 (s, 3H), 3.24-3.10 (m, 2H), 3.03-2.88 (m, 2H), 2.63-2.58 (m, 1H), 2.50-2.31 (m, 2 H), 2.28-2.12 (m, 4H), 2.03-1.75 (m, 4H), 1.17 (t, 3H, J= 1.35, 7.39 Hz); ^13^C-NMR (75 MHz, CDCl_3_, δ): 173.79, 172.52, 156.21, 136.42, 130.53, 122.27, 118.52, 109.02, 108.05, 95.34, 77.74, 77.32, 60.61, 55.83, 53.73, 51.78, 51.69, 51.22, 49.05, 34.01, 32.33, 29.55, 28.03, 24.25, 16.80, 9.16.; ESI-MS: m/z= 471.3 (M^+^ + H); Anal. Calcd. for C_26_H_34_N_2_O_6_C, 66.36; H, 7.28; N, 5.95. Found: C, 66.08; H, 7.44; N, 5.87.

#### 5-iodo-2-methoxyphenyl acetate (9)

2-Methoxyphenylacetate (28.5 g, 171.87 mmol) was added to a mixture of iodine (17.46 g, 68.75 mmol) and HIO_3_ (7.18 g, 40.8 mmol) in glacial acetic acid (190 mL), chloroform (50 mL), water (65 mL) and concentrated sulfuric acid (2 mL) and this mixture was stirred for 24 h at 40°C. Chloroform (50 mL) and water (30mL) were added and the mixture was washed with dilute NaHSO_3_ (3X) and water. The organic layer was dried with magnesium sulfate and the organic solvent was removed under vacuum to leave a residue that was recrystallized from ethanol to afford 9 as white crystals (11.7 g, 23%); (chloroform/methanol 2%, R_f_, 0.3), m.p = 75 °C; ^1^H-NMR (300 MHz, CDCl_3_, δ): 7.49-7.46 (dd, 1H J= 8.63, 2.15 Hz), 7.34 (d, 1H, J= 2.17 Hz), 6.73 (d, 1H, J= 8.66 Hz), 3.79 (s, 3H), 2.29 (s, 3H); ^13^C-NMR (75 MHz, CDCl_3_, δ): 168.58, 151.37, 140.48, 135.71, 131.62, 114.33, 81.32, 55.98, 20.55; ESI-MS: m/z= 292.9 (M^+^); Anal. Calcd. for C_9_H_9_IO_3_.0.5CH_3_COOH C, 37.29; H, 3.44. Found: C, 37.92; H, 3.09.

#### N-hydroxy-5-iodo-2-methoxybenzamide (10)

Separate solutions of hydroxylamine hydrochloride (605 mg, 8.60 mmole) in methyl alcohol (75 mL) and potassium hydroxide (1.2 g, 18.81 mmole) in methanol (50 mL) are prepared at the boiling point of the solvent. Both are cooled to 30–40 °C and the one containing alkali was added with shaking to the solution of hydroxylamine; any excessive rise of temperature during the addition is prevented by occasional cooling in an ice bath. After all the alkali has been added, the mixture is allowed to stand in an ice bath for 5 min to ensure complete precipitation of potassium chloride followed by filtration. The filtrate was added to methyl 5-iodo-2-methoxybenzoate (500 mg, 1.72 mmole) and the mixture was heated to reflux for 6 h and cooled to room temperature. The mixture was acidified with glacial acetic acid until the pH was about 6 and concentrated to remove the solvents. The residue was mixed with EtOAc (100 mL) and water (80 mL) was added to get a clear solution. The organic layer was separated and the aqueous solution was extracted with EtOAc (2 × 50 mL). The combined organic phases were washed with brine, dried over anhydrous sodium sulfate, and concentrated to afford a white solid residue, recrystallized from chloroform to afford 9 as white crystals (362 mg, 73% yield) (ethyl acetate/ hexane 1:5, R_f_ =0.3); mp = 157–158°C; ^1^H-NMR (300 MHz, DMSO, δ): 10.68 (s, 1H), 9.16 (br, s, 1H), 7.82-7.72 (m, 2H), 7.01-6.93 (d, 1H, J = 8.62 Hz), 3.81 (s, 3H); ^13^C-NMR (75 MHz, DMSO, δ): 161.50, 156.48, 139.93, 137.55, 124.89, 114.64, 82.73, 55.85; ESI-MS: m/z= 294.0 (M^+^ + 1); Anal. Calcd. for C_8_H_8_INO_3_ C, 32.79; H, 2.75; N, 4.78. Found: C, 32.82; H, 2.61; N, 4.79.

#### N-hydroxy-4-iodo-2-methoxybenzamide (11)

This compound was prepared using the same procedure for 7 with methyl-4-iodo-2-methoxybenzoate as substrate to afford an off-white solid that was recrystallized from ethyl acetate/n-hexane to yield 10 as off-white crystals (102 mg, 21%); (ethyl acetate/ hexane 1:5, R_f_ =0.4 ); mp = 105–106°C; ^1^H-NMR (300 MHz, CDCl_3_, δ): 10.24 (s, br, 1H), 7.82 (d, 1H, J = 8.23 Hz), 7.41 (d, 1H, J = 8.12 Hz), 7.28 (s, 1H), 3.95 (s, 3H); ^13^C-NMR (75 MHz, CDCl_3_, δ): 163.13, 157.03, 132.89, 130.94, 120.77, 118.01, 99.66, 56.52; ESI-MS: m/z= 294.0 (M^+^ + 1); Anal. Calcd. for C_8_H_8_INO_3_.0.1CH_3_COOC_2_H_5_ C, 33.42; H, 2.94; N, 4.64. Found: C, 33.48; H, 2.76; N, 4.57.

### General procedure for synthesis of rescinnamine derivatives (12–15)

A similar Heck coupling procedure without a phosphine ligand using the appropriate substituted aryl iodides affords the corresponding substituted rescinnamine derivatives (12–15).

#### For 12

Yellowish brown solid recrystallized from diethyl ether, yield = 132 mg (35%), (chloroform/methanol 2%, R_f_ = 0.2), mp = 202–203 °C; ^1^H NMR (300 MHz, CDCl_3_, δ): 7.66 (s, 1H), 7.63 (d, J = 15.91 Hz, 1H), 7.32 (d, J = 8.54 Hz, 1H), 6.94-6.90 (m, 2H), 6.82 (s, 1H), 6.74 (d, J = 8.70 Hz, 2H), 6.27 (d, J = 15.88 Hz, 1H), 4.96-4.87 (m, 1H), 4.44 (s, 1H), 3.88 (s, 3H), 3.83 (s, 3H), 3.81 (s, 3H), 3.51 (s, 3H), 3.42-3.44 (m, 1H), 3.20-3.15 (m, 2H), 3.04-2.89 (m, 2H), 2.73-2.61(m, 1H), 2.50-2.42 (m, 2H), 2.29-2.17 (m, 2H), 2.05-1.77 (m, 4H), 1.28-1.18 (m, 2H); ^13^C NMR (75 MHz, CDCl_3_, δ): 172.92, 166.68, 156.23, 149.42, 145.33, 136.54, 136.38, 130.51, 127.47, 122.21, 120.30, 118.53, 115.41, 113.13, 110.18, 109.05, 108.06, 95.23, 77.88, 65.84, 60.75, 55.83, 55.59, 53.75, 51.78, 51.21, 49.08, 34.04, 32.32, 29.74, 24.27, 16.81, 15.26; ESI-MS: m/z= 590 (M^+^ + 1), 295 (M^+^ + 2). HRMS-ESI^+^ (m/z): [M + H]^+^ calcd for C_33_H_40_N_3_O_7_, 590.2866; found, 590.2846; Anal. Calcd for C_33_H_39_N_3_O_7_.0.8H_2_O: C, 65.61; H, 6.77; N, 6.96; Found: C, 65.27; H, 6.63; N, 6.87.

#### For 13

Pale yellow flakes recrystallized from diethyl ether, yield = 153 mg (41%), (chloroform/methanol 2%, R_f_ = 0.4), mp = 210–213 °C; ^1^H NMR (300 MHz, CDCl_3_, δ): 7.81 (s, br, 1H), 7.61 (d, J = 15.8.4 Hz, 1H), 7.31 (d, J = 8.54 Hz, 1H), 7.01 (d, J = 7.98 Hz, 1H), 6.96 (s, 1H), 6.82 (s, 1H), 6.75 (d, J = 8.54 Hz, 1H), 6.66 (d, J = 7.97 Hz, 1H), 6.23 (d, J = 15.83 Hz, 1H), 4.95-4.80 (m, 1H), 4.43 (s, br, 1H), 4.14 (s, 1H), 3.88 (s, 3H), 3.82 (s, 3H), 3.80 (s, 3H), 3.50 (s, 3H), 3.18-3.13 (m, 2H), 3.03-2.93 (m, 2H), 2.67-2.58 (m, 1H), 2.49-2.40 (m, 2H), 2.27-2.16 (m, 2H), 1.92-1.77 (m, 5H); ^13^C NMR (75 MHz, CDCl_3_, δ): 172.99, 166.94, 156.15, 146.97, 145.77, 139.20, 136.36, 130.51, 128.67, 124.60, 123.34, 122.15, 118.52, 114.06, 113.39, 108.98, 107.96, 95.19, 60.77, 55.82, 55.50, 53.73, 51.82, 51.19, 49.03, 41.81, 34.01, 32.30, 30.95, 29.77, 24.24, 16.78, 13.85; ESI-MS: m/z= 590 (M^+^ + 1), Anal. Calcd for C_33_H_39_N_3_O_7_.0.6H_2_O: C, 66.01; H, 6.75; N, 7.00; Found: C, 66.11; H, 6.64; N, 6.71.

#### For 14

Grayish white solid, yield = 289 mg (70%), recrystallized from benzene, (chloroform/methanol 6%, R_f_ = 0.3), mp = 245–255 °C; ^1^H NMR (500 MHz, DMSO, δ): 10.96 (s, 1H), 7.57 (s, 1H), 7.49 (d, 1H, J= 15.78 Hz), 7.37-7.34 (m, 2H), 7.23 (s, 1H), 6.86 (s, 1H), 6.71 (d, 1H, J= 8.49 Hz), 6.27 (d, 1H, J= 15.79 Hz), 5.06 (s, br, 1H), 5.06 (s, 1H), 4.69-4.72 (m, 1H), 3.81 (s, 3H), 3.79 (s, 3H), 3.77 (s, 3H), 3.74-3.68 (m, 2H), 3.65-3.57 (m, 1H), 3.44 (s, 3H), 3.17-3.07 (m, 1H), 2.84-2.99 (m, 1H), 2.85-2.82 (m, 1H), 2.73-2.69 (m, 1H), 2.30-2.08 (m, 5H), 1.94-1.83 (m, 2H); ^13^C NMR (125 MHz, DMSO, δ): 171.76, 171.24, 165.79, 157.77, 155.92, 149.02, 147.59, 145.74, 137.31, 128.29, 124.66, 122.56, 120.84, 120.64, 118.63, 118.35, 112.76, 112.08, 109.04, 105.34, 94.82, 77.12, 75.66, 60.23, 55.66, 55.25, 54.32, 51.95, 50.16, 31.27, 30.34, 28.34, 22.49, 15.49; ESI-MS: m/z= 635.2 (M^+^ + H); HRMS: [M ^+^+ H- CH_3_] calcd for C_34_H_39_N_2_O_10_, 635.2605; found, 635.2582; Anal. Calcd for C_35_H_40_N_2_O_10_.2.5H_2_O: C, 61.71; H, 6.25; N, 4.23; Found: C, 61.84; H, 5.87; N, 4.28.

#### For 15

Dark yellow solid, yield = 140 mg (35%), recrystallized from diethyl ether, (chloroform/methanol 5%, R_f_ = 0.1), mp = 229–231 °C; ^1^H NMR (300 MHz, DMSO, δ): 10.49 (s, 1H), 8.96 ( s, br, 1H), 7.63 (d, 1H, J= 15.81 Hz), 7.20 (d, 1H, J= 8.57 Hz), 7.06 (s, 2H), 6.80 (s, 1H), 6.62-6.54 (m, 2H), 4.89-4.76 (m, 1H), 4.35 (s, 1H), 3.82 (s, 6H), 3.79 (s, 3H), 3.75 (s, 3H), 3.42 (s, 3H), 3.10-2.97 (m, 3H), 2.89-2.79 (m, 3H), 2.69-2.60 (m, 3H), 2.42-2.14 (m, 5H); ^13^C NMR (75 MHz, DMSO, δ): 171.61, 165.98, 155.05, 148.01, 145.61, 138.35, 136.35, 131.20, 128.52, 124.37, 121.65, 117.81, 114.96, 108.01, 106.32, 105.92, 94.77, 77.54, 76.40, 64.87, 60.05, 56.09, 55.18, 53.32, 51.74, 51.05, 50.74, 48.62, 33.19, 32.14, 29.53, 23.46, 16.44, 15.12; ESI-MS: m/z= 621.3 (M^+^ + H).

Molecular Dockin-The molecular docking was performed in four phases; structural model generation, ligand library generation, receptor grid generation and finally docking of the ligand library into the receptor grid. Models for the structures were generated using molecular dynamics as in our previous works (Vasilyeva, A et al, 2009; Vasilyeva, A et al, 2010;Salsbury. 2-1-). However, for this work more extensive simulations were used. The details of these simulations are reported elsewhere (Negureanu and Salsbury, 2012). In short, the simulations were four 20ns NPT all-atom simulations based on the human MSH2/6 crystal structure (Morris et al, 2009), with the (1,2)G crosslink, which is the predominate damage due to cisplatin. The structure selected was the median structure of the most populated cluster found from all-atom RMSD-based clustering (Negureanu and Salsbury, 2012). The appropriate pdbqs file was generated with the DNA removed, so that just the protein remained, using defaults from Autodock4.

Libraries for docking were based on the core of the rescinnamine structure (Figure 7). All possible derivatives were made based on this structure (Figure 7), with R1, R2 and R3 selected from H, OH, COOH, NH_2_, and OCH_3_. Also, a structure without the modified phenyl ring was docked (Compound 1, Table 1). The best of these compounds were suggested for chemical synthesis based on their synthetic accessiblity. We also explored larger libraries, and ones with other functional modifications, but these were not readily synthesized and not discussed herein. Autodock tools were used to generate 3D pdbq files with charges and the correct number of rotatable bonds.

The grids for docking were generated using Autodock4 with the grid centered at the position of the platinum atom in the full protein-DNA complex, however, the DNA was removed prior to grid generation. Cubic 22.5A grids were generated for electrostatics, and vdW parameters for C, S, O, N and polar H with a grid spacing of 0.375A.

The dockings were performed using Autodock4’s defaults for it’s Lamarckian Genetic Algorithm with a population size of 150, a maximum number of energy evaluations of 5 million and a maximum number of generations of 27000. Each derivative was subjected to 256 runs and ranked according to the best predicted Ki.

## RESULTS

### Rescinnamine as a first lead in inducing MSH2-dependent cell death

**Fig. 1:**
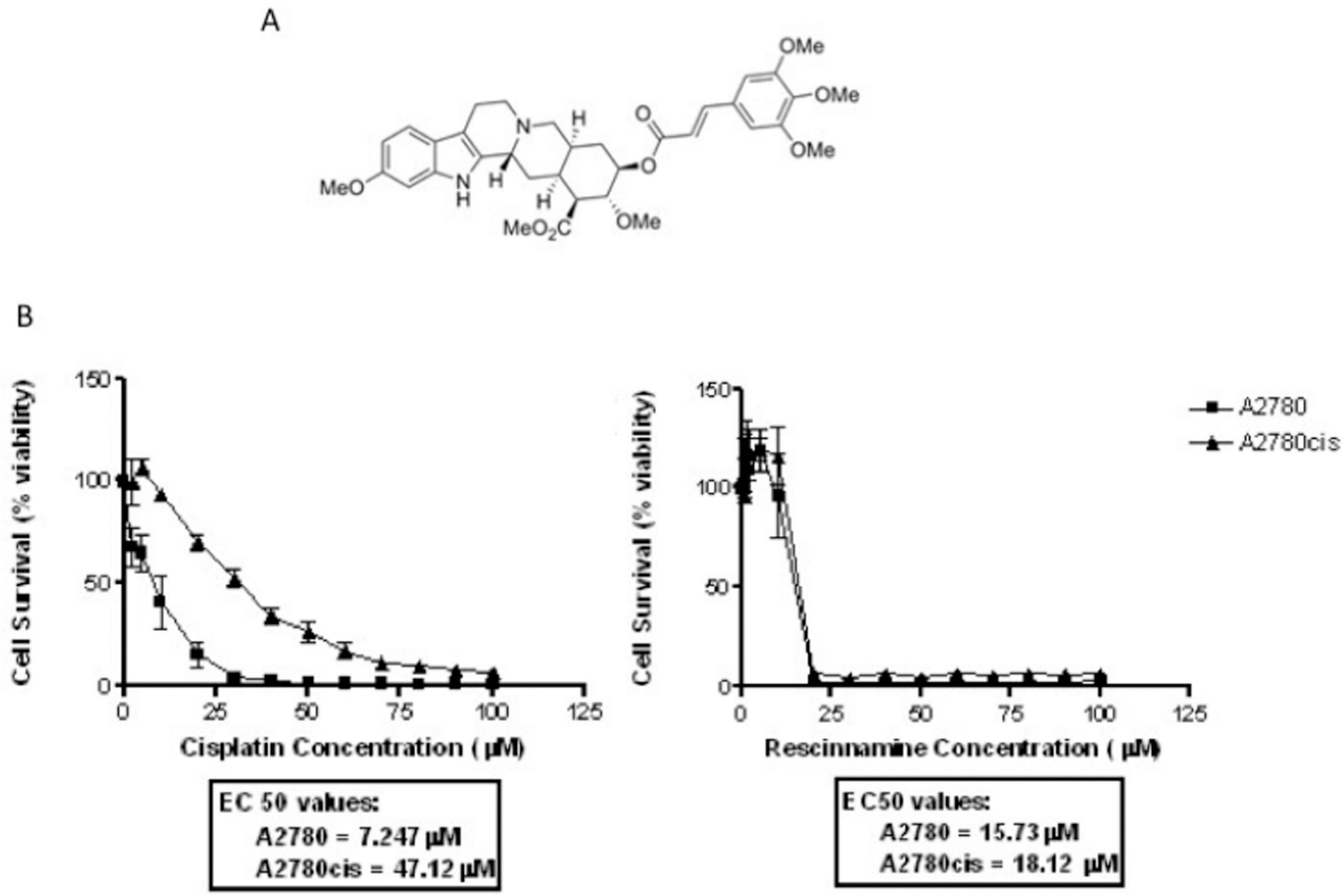
Effect of rescinnamine on viability of cisplatin-resistant cells. A: Structure of rescinnamine. B: The ovarian cancer cell line A2780 and its cisplatin-resistant derivative were exposed to increasing concentrations of cisplatin (left) and rescinnamine (right). Cell survival was determined in relation to untreated cells.

We had previously shown that reserpine and rescinnamine are capable of inducing cell death in an MSH2-dependent manner (Vasilyeva 2009, 2010). Further experiments demonstrated that this indole alkaloid is capable of overcoming resistance to cisplatin in ovarian cancer cells (Fig. 1).

**Fig. 2:**
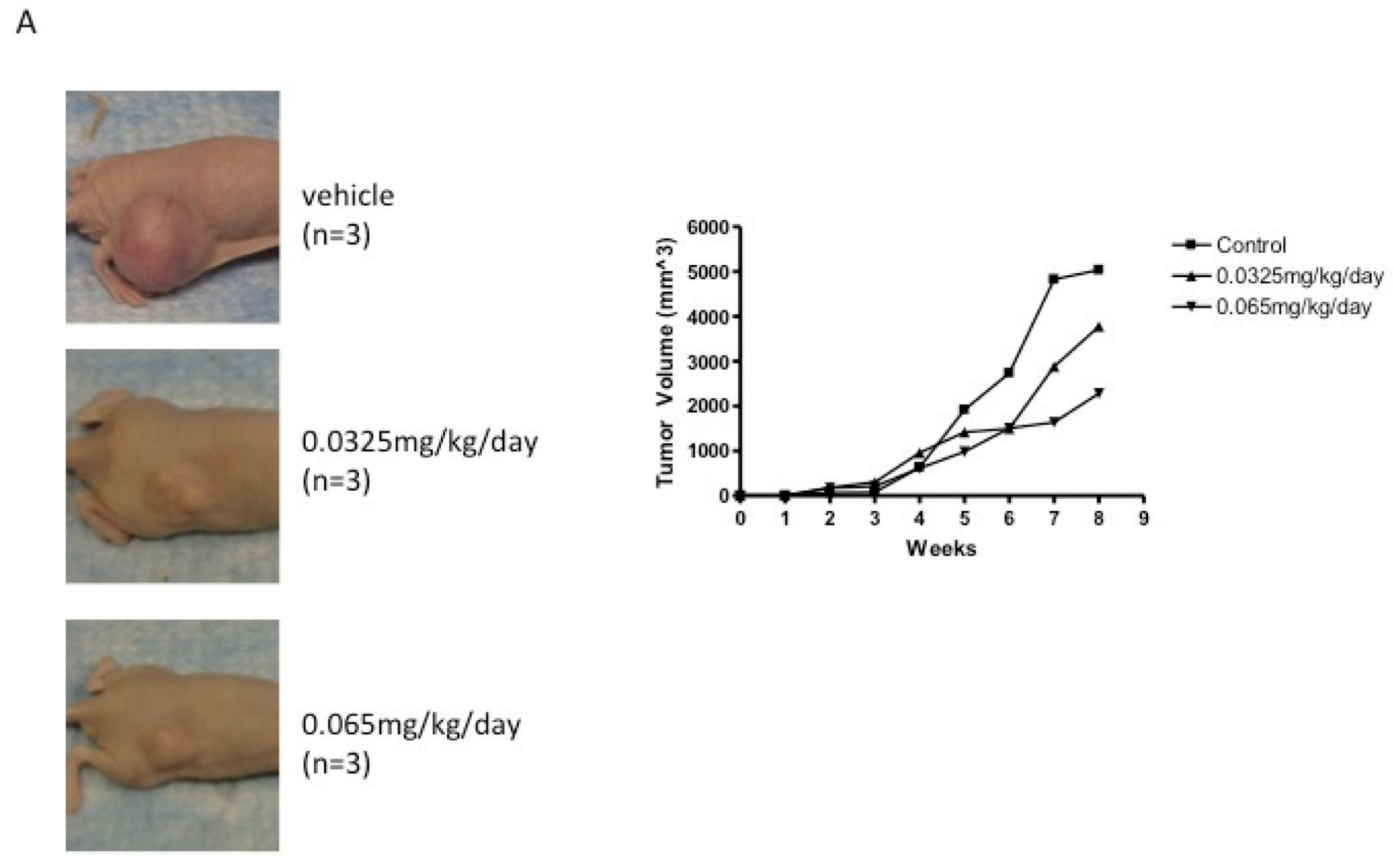
Effect of rescinnamine on tumor growth in a mouse xenograft model. A: Images of mice untreated and treated with several different concentrations of rescinnamine. Tumors were obtained by injection with the colorectal cancer cell line SW620. B, C: Graphs illustrating tumor growth and body weight of untreated and treated mice in two different cell systems (colorectal cancer (B), endometrial cancer (C)).

These initial results suggested that computational modeling of docking to a specific target protein can identify lead compounds that have specific characteristics. In our case, rescinnamine was identified to be a good lead toward the development of an effective cancer drug that targets a very specific, pro-apoptotic mechanism that is more prevalent in cancer cells. When applied to a xenograft model, however, the hypotensive activity of the drug prevented administration of statistically effective doses, though a significant trend toward tumor inhibition is observed (Fig. 2). Since none of the commercially available reserpine analogs showed much promise in inducing MSH2-dependent cell death (Vasilyeva et al., 2010), we engaged computational modeling and chemical synthesis to generate novel rescinnamine analogs.

In keeping with our previous studies, we computationally-modeled derivatives of rescinnamine for their interaction with human MSH2/6. MSH2/6 were previously shown to have pro-apoptotic function; cancer specificity is achieved by elevation of these proteins in cancer cells. In a targeted approach, computational studies were used to determine if the activity of rescinnamine could be improved by modification of functional groups on the ring systems, or alternatively not perturbed while perturbing the hypotensive effects of rescinnamine. In this work, the former, changes in activity, is explored. Although many possible functionalization sites exist in rescinnamine with many possible R-groups, we chose to limit our exploration to positions R_1_, R_2_ and R_3_ and to H, OH, COOH, NH_2_, and OCH_3_ as possible R groups (Figure 7). For rescinnamine, R_1_, R_2_ and R_3_ = OCH_3_, and such an approach avoids a combinatorial explosion computationally yet systematically varies structure in an area that clearly effects biological outcomes (Vasilyeva 2009, 2010). In addition, we restricted ourselves to derivatives that were synthetically feasible and this combination of computational prediction and synthetic accessibility defined 1–8 and 12–15 as a logical and practical initial group of compounds to determine the structure activity relationships of rescinnamine.

#### Organic Synthesis of rescinnamine analogs

Promising lead molecules that were predicted to induce MSH2-dependent cell death and had a favorable partition coefficient were considered for chemical synthesis (Table 1).

**Fig. 3:**
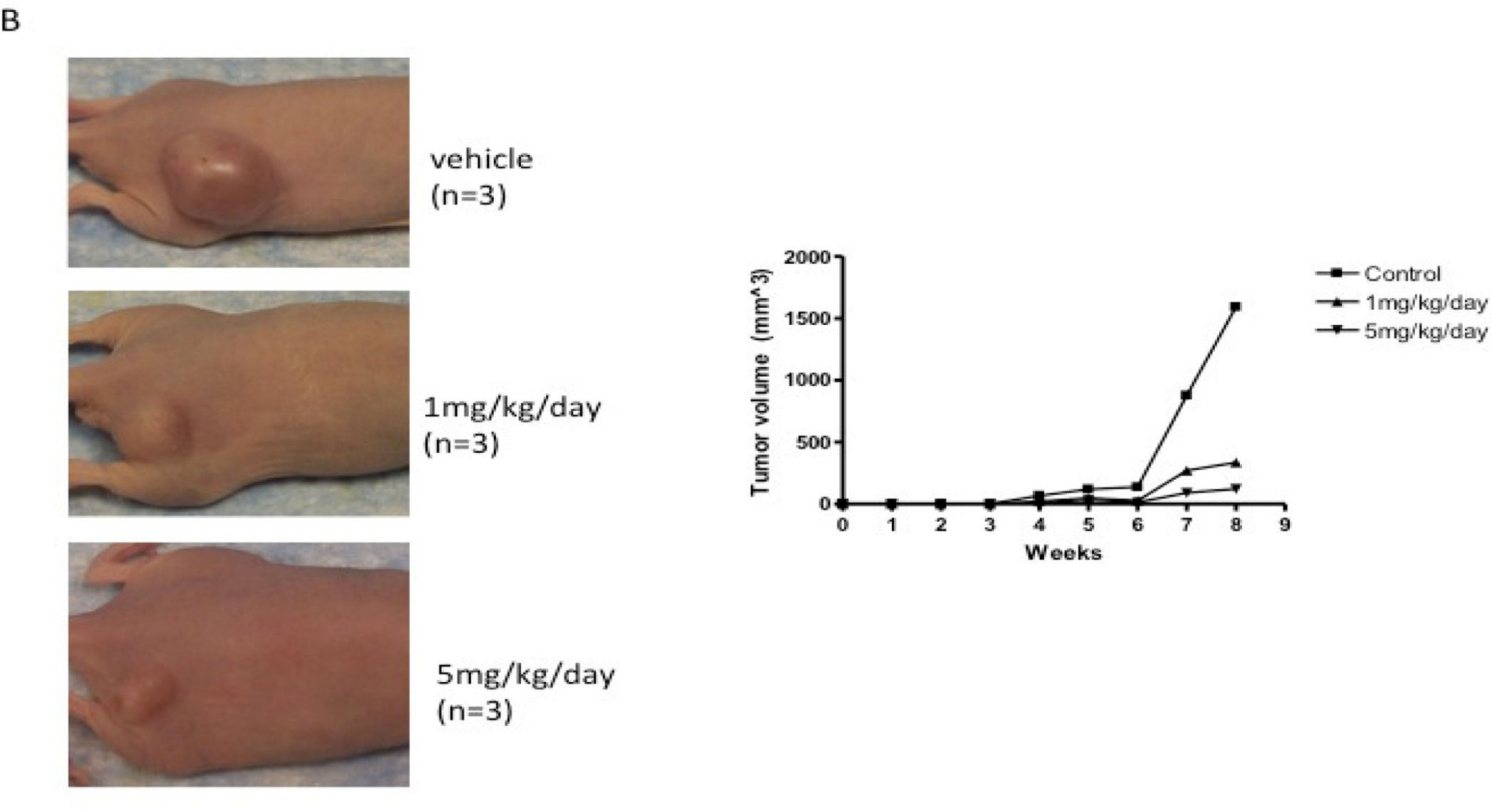
Synthetic procedures for the preparation of rescinnamine derivatives (1–8). Condensation reactions between methyl reserpate and acyl chlorides to give 1 and 8. Heck coupling procedure with 1 to yield rescinnamine derivatives 2–7.

Treatment of methyl reserpate, formed by the basic methanolysis of reserpine (Vasilyeva et al., 2010), with acryloyl chloride yields acryloyl reserpate (1) in 35% yield (Figure 3) (Pearce et al., 1989). Similar treatment of 1 with propionoyl chloride yields the saturated derivative 8 (Figure 3). Exposure of 1 to various commercial or synthetic substituted aryl iodides (9) to palladium catalyzed Heck coupling conditions gives the disubstituted rescinnamine derivatives (2–7) in 27–68% yield and coupling constant analysis shows the exclusive formation of the trans stereoisomer (Figure 3) (Ziegler et al., 1978)

**Fig. 4:**
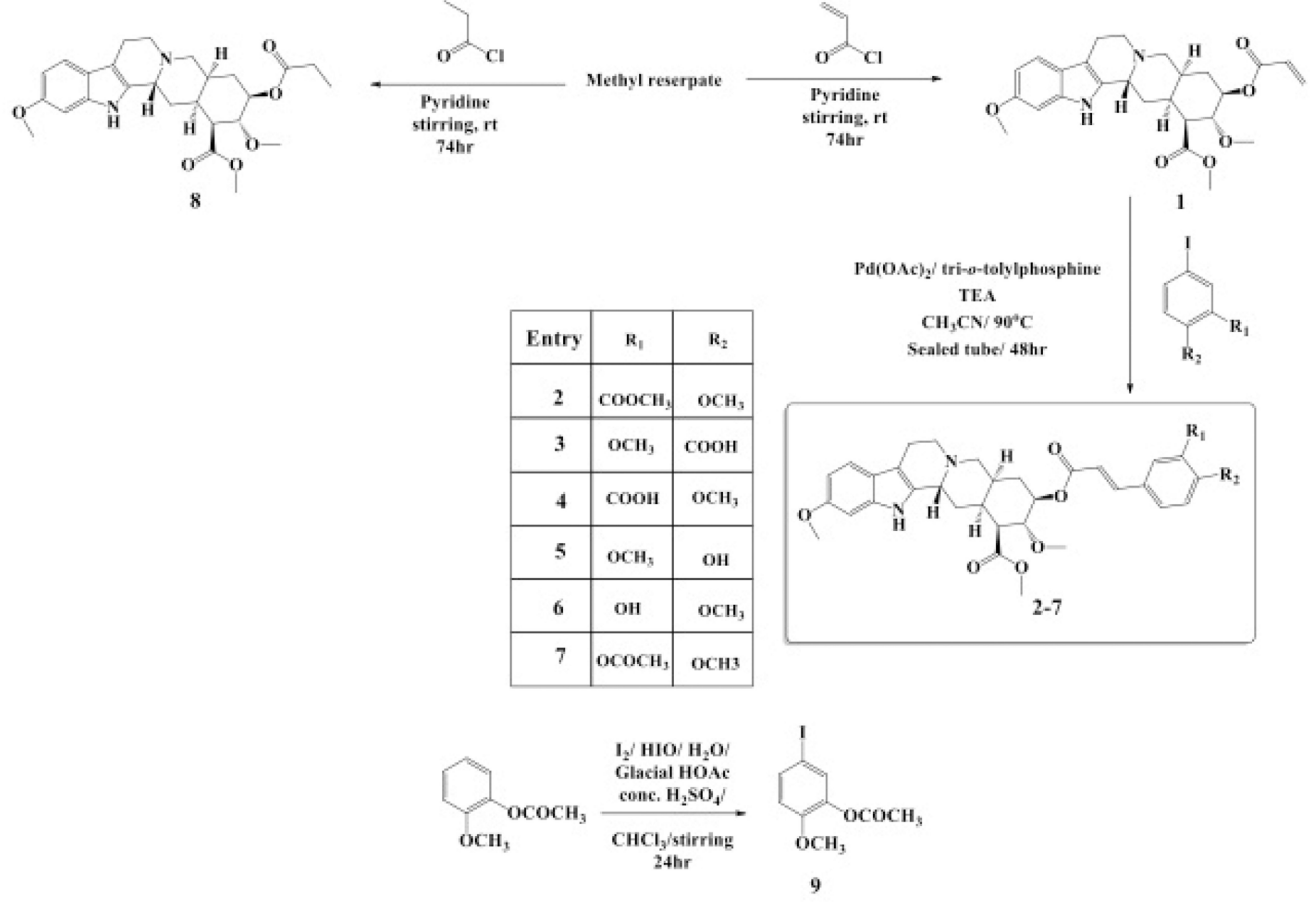
Synthetic procedures for the preparation of rescinnamine derivatives (12–13). Heck coupling of synthetic hydroxamic acid aryl iodides gives the amino-substituted rescinnamine derivatives 12 and 13.

Condensation of the commercially available methyl esters with hydroxylamine produces the corresponding hydroxamic acids (10 and 11, Figure 4) (Hoshino et al., 2009) and Heck coupling of 1 to 10 and 11 in the absence of a phosphine ligand yields the aniline derivatives of rescinnamine (9 and 10, exclusive E stereochemistry) in 35 and 41% yield, respectively rather than the expected hydroxamic acids (Figure 4). (Patel et al., 1977)

**Fig. 5:**
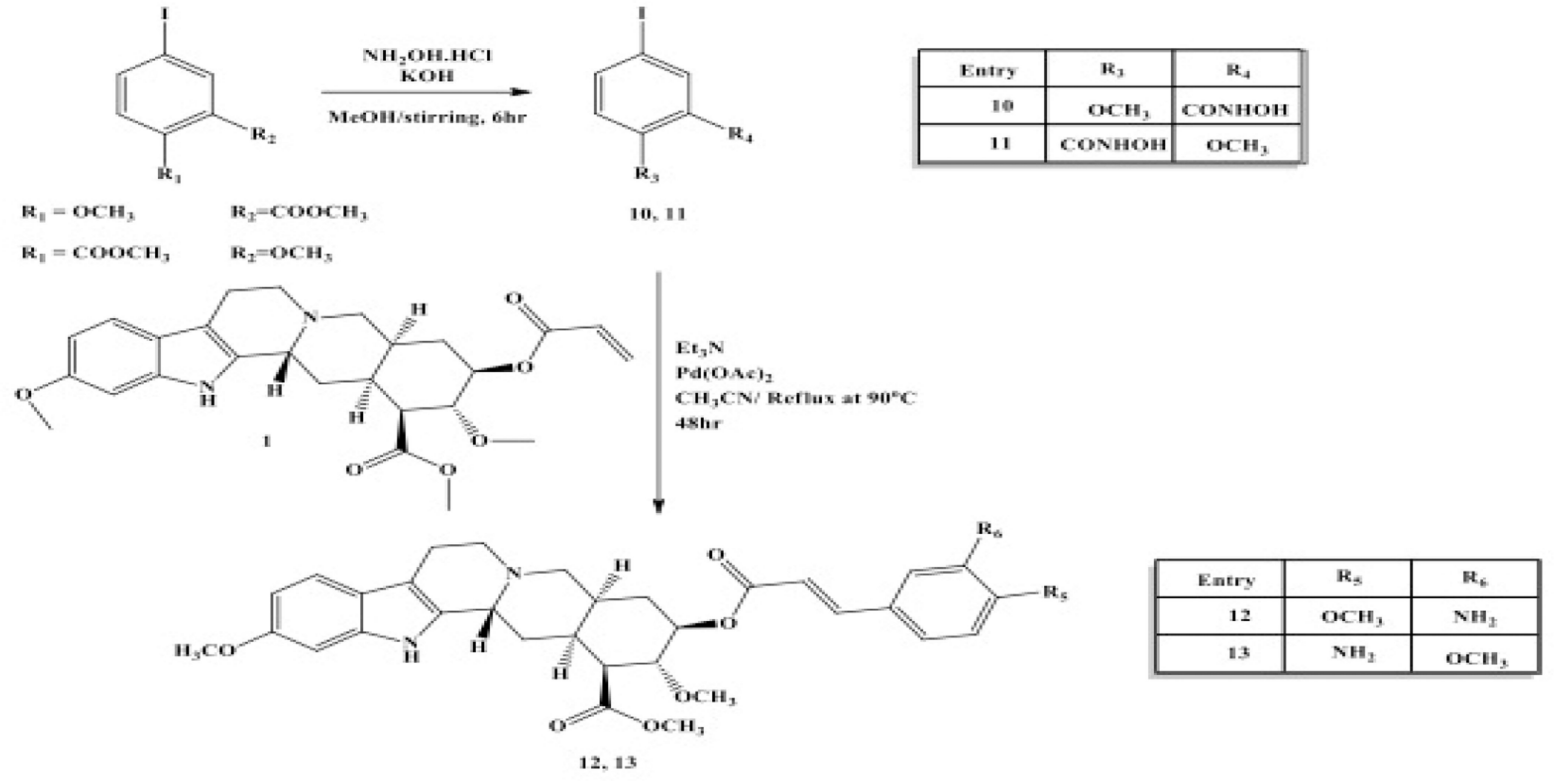
Synthetic procedures for the preparation of rescinnamine derivatives (14–15). Heck coupling of commercially available aryl iodides to give rescinnamine derivatives 14 and 15.

Such results suggest either a base or palladium-mediated Lossen rearrangement of these hydroxamic acid substrates, a process that holds some literature precedence (Hoshino et al., 2009). Similar Heck coupling of 1 to commercial aryl iodides yields the tri-substituted rescinnamine derivatives 14 and 15 in 70 and 35% yield, respectively (Figure 5). Extensive mass spectrometry and two-dimensional nuclear magnetic resonance (NMR) experiments confirm the structure of 14, which lacks the expected p-methoxy group of the starting material (Figure 5).

A combination of proton and carbon NMR spectroscopy, mass spectrometry and elemental analysis confirms the identity of 1–15.

#### Effects of rescinnamine analogs on cell viability

**Fig. 6:**
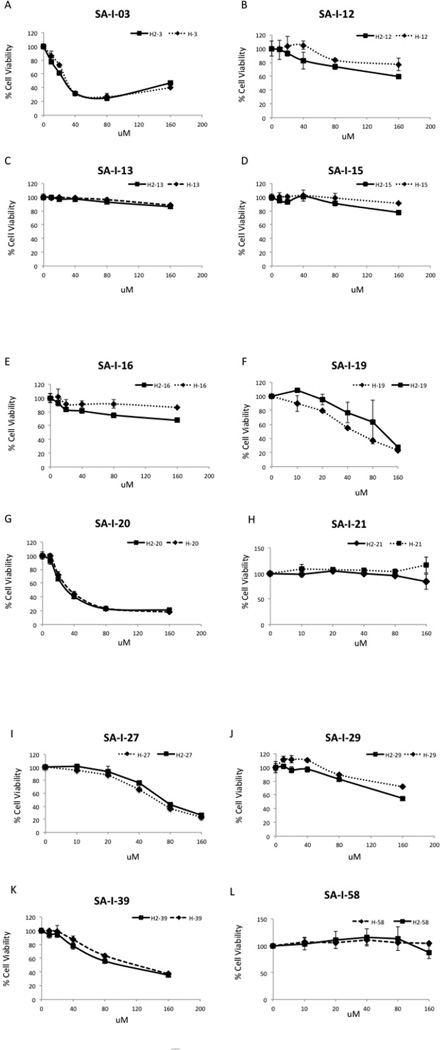
Cell Survival of MSH2 proficient and deficient cells after treatment with rescinnamine analogs. HEC59 and its isogenic cell line containing a chromosome 2 transfer were treated with increasing concentrations of the indicated compound (Table 1). Cell viability is graphed in dependence of concentration.

We next tested these new rescinnamine analogs in a well-defined cellular system with an endometrial cell line deficient (HEC59) and proficient (via chromosome transfer, HEC59 + chr.2) for MSH2. This cell system allows to determine whether our new analogs hit their target and, generally, induce cell death. The assays identified a few compounds that induced cell killing in the micromolar range (Fig. 6 and Table 1, compounds 1, 6, 7, 13, 15 and to a lesser extent 12).

However, little significant MSH2-dependence was observed for these compounds. The only compounds that demonstrated a small difference between MSH2-proficiency and deficiency were 2, 5 and 13 (Figure 6 and Table 1). No significant difference was detected when determining the IC50 values and these results are surprising in-light of our previous work. Previous studies have suggested that in addition to MSH2-dependent cell-killing due to rescinnamine and derivatives, there is off-target cell-killing as well (Vasilyeva et al., 2010). These results suggest considerable off-target killing for these classes of compounds.

## DISCUSSION

**Fig 7:**
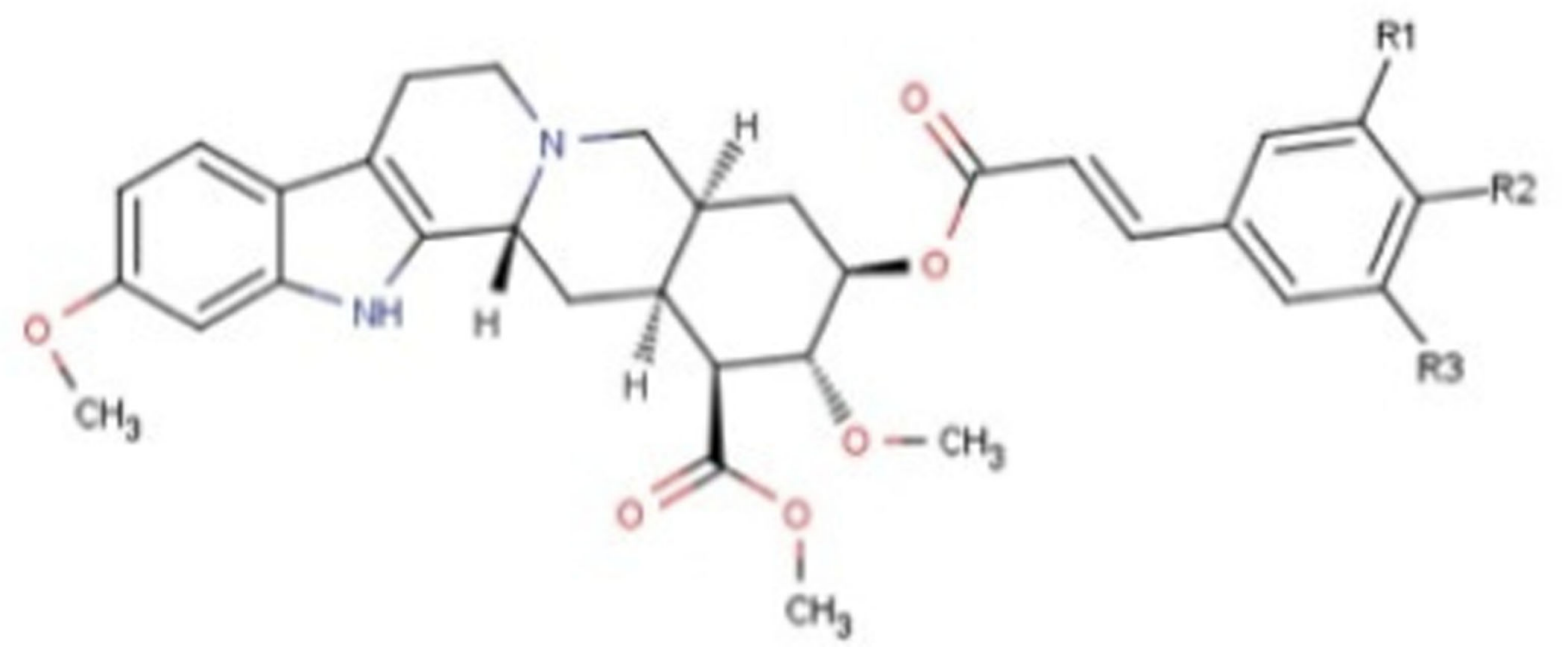
Structure of Rescinnamne with sites for computational modification.

We here describe an interdisciplinary approach involving computational simulation, chemical synthesis and cell biology to modify a lead compound, rescinnamine, to target a specific pro-apoptotic mechanism in cancer cells. Such approach, in general, will allow personalized treatment development and can be applied to different target molecules. In our study, we chose the mismatch repair protein MSH2 as an unusual target, as it only previously was identified for its pro-apoptotic activity that appears to be specific to certain mechanisms and drugs, and therefore represents an important, yet challenging target for cancer therapy. Here, computational modeling studies indicate that rescinnamine derivatives containing H, OH, COOH, NH_2_, and OCH_3_ groups in positions R_1_, R_2_ and R_3_ (Figure 7) will likely demonstrate MSH2-dependent cellkilling.

For rescinnamine, R_1_, R_2_ and R_3_ = OCH_3,,_ a polar electron-donating group that does not contain a hydrogen bond donor or any specific acidic group. Introduction of these other groups allows for a wide variance of the structural and electronic properties to observe the effect on activity. The OH and NH_2_ groups will both be electron donating and contain hydrogen bond donors with the phenol group being slightly acidic and the COOH group will be electron withdrawing and acidic. This array of substitutions in different positions provides the first structure activity relationship regarding the MSH2-dependent cell killing actions of rescinnamine.

Chemically, the target compounds (2–7) and (12– 15) were prepared by a palladium-mediated Heck coupling of acryolyl reserpate (1) and various aryl iodides. To the best of our knowledge, this work demonstrates the first preparation of rescinnamine derivatives using this organometallic method and further shows the wide applicability of palladium-based chemistry even in complex natural products containing multiple functional groups. Given the large number of available aryl iodides and the straightforward preparation of 1, this method provides an attractive convergent alternative to the classic preparation of rescinnamine derivatives through the acylation of methyl reserpate with various cinnamoyl chlorides. This method may prove useful for generating other specific rescinnamine derivatives for these studies.

In terms of biological activity a number of points can be made from these studies. Surprisingly, compound 1, the synthetic intermediate that lacks the extra ring system, demonstrates activity (not MSH2-dependent). However, the saturated version of 1 (compound 8) does not lead to cell killing suggesting that 1 may act through an alternative mechanism compared to rescinnamine. Compound 1 contains an unsaturated acrylic ester group that suggests the possibility of Michael reactions with biological nucleophiles could form the basis of this non-MSH2-dependent activity.

As noted, this work only identified a few compounds that induced cell killing in the micromolar range (Fig. 6 and Table 1) and little significant MSH2-dependence was observed for these compounds. One general observed trend suggests that replacement of the OCH_3_ group at R_2_ with an acidic hydrogen bonding group greatly diminishes activity (2, 4, 6, 7 (with OCH_3_) vs 3, 5, 14). This trend suggests the acidic OH and COOH groups, which likely exist in an anionic form, result in an unfavorable interaction with the protein. The amine substituted compounds 12 and 13, which should be neutral or positive under physiological conditions, did not show this difference supporting the notion that acidic groups at R_2_ diminish activity. A closer examination of Table 1 shows the drastic difference between isomers, 3 vs 4 especially, and also to a lesser event 12 vs 13. This difference also suggests a preference for electron donating and non-acidic groups at R2 to avoid a reduction in IC50 when MSH2 is present (as noted above). In 4 there is an electron donating group (OCH3) at R2, and an electron withdrawing group at R1. In 3, these groups are switched and there is an ∼8-fold change in sensitivity to MSH2. In 13 and 12, there are electron donating groups at both R1, and R2, but when the stronger electron donor is at R2, there is nearly a 2-fold change in sensitivity to MSH2. The only compounds that demonstrated a small difference between MSH2-proficiency and deficiency were 2, 5 and 13 (Figure 6 and Table 1).

Taken together, these results demonstrate that the nature of functional groups in key positions of rescinnamine is critical to activity as altering these functional groups can lead to MSH2-independent cell-killing, or even to reduction in IC50 when MSH2 is present, such as with 3 and 12. These compounds are of particular interest because the difference suggests some sort of interaction or effect due to MSH2, but one that does not lead to cell death.

In conclusion, cell death initiation itself, and more so dependence on MSH2, is largely dependent on specific functional groups, rather than just the presence of ring systems of rescinnamine. This first examination of the structure activity relationship of rescinnamine shows that small changes to the structure of rescinnamine affect its activity on cell death significantly, which represents a great challenge to further work in developing derivatives of rescinnamine. However, this work does suggest three possible avenues for further research. First, a search for the off-targets that are being affected so as to design away off-target interactions. Second, a study as to why MSH2 desensitizes cells to two compounds (3 and 12), which may be possible candidates for further study, if they interact with MSH2, but not in a manner to promote cell death. Third, an exploration of other functional sites away from the phenyl ring for modification.

## Figures and Tables

**Table 1:** Rescinnamine Analogs IC50 values for individual compounds. Where appropriate, graphs that did not reach 50& mortality were extrapolated.

			IC50	
Entry	Compound Number	Compound	MSH2 deficient	MSH2 proficient
	1	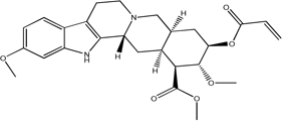	20.7	20.37
	8	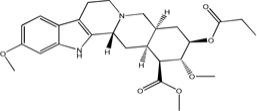	NA	NA
	2	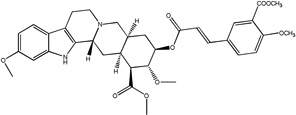	50.72	48.91
	3	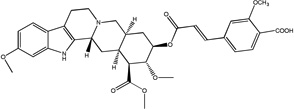	163.1	1223
	4	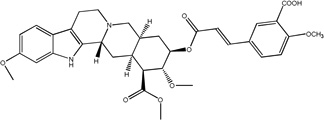	89.29	84.23
	5	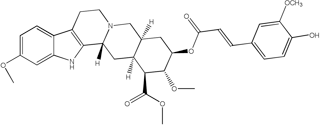	NA	NA
	6	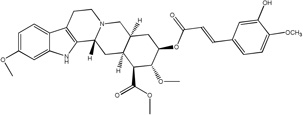	22.32	21.29
	7	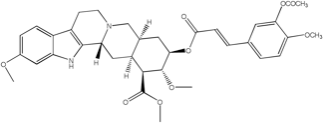	39.28	NA
	14	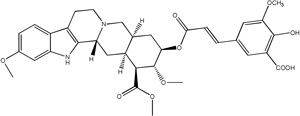	NA	NA
	15	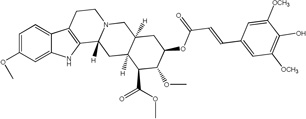	47.67	54.53
	12	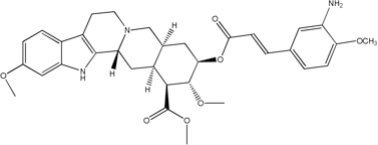	77.25	113.4
	13	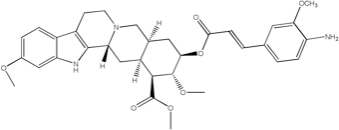	85.73	68.38

NA: not applicable, since data did not reach below 50%.
